# Supplementary material for: IKK-β inhibitor TPCA-1 increases Gd-IgA1 production and decreases C1GALT1 expression in EBV-immortalized B cells from human donors
Source: Front Immunol. 2026 Oct 28;17:1913226. doi: 10.3389/fimmu.2026.1913226 (PMC13752088; doi:10.3389/fimmu.2026.1913226)
Supplement: Supplementary file 1 [file SupplementaryFile1.docx]

**Supplementary figures and tables**

**Supplementary Figure 1: Gating strategy for SSC-based subpopulations.** Left, single cells were identified on FSC-A versus FSC-H. Center, gates for the SSC-low and SSC-high subpopulations on BSSC-A distribution for control (top) and TPCA-1 48 hr treated (bottom). Gating is based on bottom 20% and top 20% of control. Right, representative half-offset overlay of IgA1 (PE) fluorescence for the four resulting subpopulations: TPCA-1 SSC-high (green), TPCA-1 SSC-low (orange), control SSC-high (red) and control SSC-low (blue). BSSC-A is the side-scatter parameter as designated by the Agilent Opteon and corresponds to SSC-A elsewhere in this manuscript.

**Supplementary Figure 2: Dose response for TPCA-1 by EBV-immortalized B cells.** Cells were plated at 1x10^6^/mL. **A)** Cell viability expressed as percent of sample after 48 hr. **B)** Cell count (x10^6^) after 48 hr. **C)** Cell viability, percent change from control after 48 hr. **D)** Cell number, percent change from control after 48 hr. Vehicle is DMSO at the same concentration as the respective TPCA-1 treatment. Ctrl = control. ***p = 0.01.**

**Supplementary Figure 3: BAY11 inhibitor impacts Gd-IgA1 and IgA1 in a similar manner as TPCA-1.** Four EBV-immortalized IgA1-secreting B-cell lines were treated for 48 hr with BAY11. **A)** Degree of galactose deficiency of secreted IgA1 (expressed as U of Gd-IgA1 per 100 ng IgA1) increased in 4/4 cell lines (geometric mean 1.39×, p = 0.076). Lines connect paired samples. **B)** Total secreted IgA1 decreased in 4/4 cell lines (geometric mean 0.88×, p = 0.047). Lines connect paired samples. **C)** log₂ fold change vs. untreated controls for both readouts; horizontal bars represent mean values. Each point represents an individual cell line. Paired t-test for log₂-transformed values, 2-tailed. n = 4. Abbreviations: Ctrl = control; BAY11 = BAY11-7082.

**Supplementary Figure 4: IgA1 and Gd-IgA1 inter-condition correlations across cell lines.** IgA1 production (top row) and Gd-IgA1 production (bottom row) were compared across control, vehicle, and TPCA-1 conditions. Each data point represents an individual donor. Dashed line represents linear fit. Spearman correlation coefficients (ρ) and p-values are shown for each comparison. IgA1 production was significantly correlated across all condition pairs (top row), while Gd-IgA1 production showed no significant correlation between any condition (bottom row). IgA units (ng/mL), Gd-IgA1 units expressed relative to the Gd-IgA1 standard (U). TPCA = TPCA-1.

**Supplementary Figure 5: Effects of TPCA-1 on the phosphorylation of IKKβ, ERK 1/2, and STAT3.** **A)** IKKβ (open circles) and pERK 1/2 (filled circles) average median fluorescence intensity (MFI) in control and TPCA-1-treated cells at 1 hr. TPCA-1 decreased IKKβ (p < 0.01) and decreased pERK 1/2 (p = 0.02). **B)** pSTAT3 (Y705) average MFI in control and TPCA-1-treated cells at 1 hr (p < 0.01). Bars represent mean +/- SD, n = 6. *= p < 0.01. **= p < 0.02.

**Supplementary Figure 6: Characterization of SSC subpopulations at 1 hr. A)** HPA reactivity (lectin specific for unmodified GalNAc) in high- and low-SSC-A subpopulations across control, vehicle, and TPCA-1 conditions. **B)** Frequency of high- and low-SSC-A subpopulations as percent gated from parent across treatment conditions. **C)** Percent change from control in high- and low-SSC-A subpopulations after vehicle or TPCA-1 treatment. **D)** p65 NF-κB (S536) mean fluorescence intensity in high- and low-SSC-A subpopulations across treatment conditions. Data points represent individual samples. Bars represent mean +/- SD. TPCA = TPCA-1.

**Supplementary Figure 7: Baseline immunophenotype of HPA^+^ and HPA^-^ subpopulations.** Untreated EBV-immortalized B cells from eight donors were stained with a 15-marker B-cell panel and gated on live, single cells into HPA^+^ (filled circles) and HPA^-^ (open circles) subpopulations; each pair of connected points represents one donor cell line. **A)** SSC-A mean: HPA^+^ cells were more granular than HPA^-^ cells from the same sample (1.33-fold, p = 0.0004; higher in 8/8 lines). **B)** FSC-A mean: cell size did not differ (0.95-fold, p = 0.69; 4/8 lines). **C)** CD138 median fluorescence intensity (MFI) (5.07-fold, p = 0.0002; 8/8 lines). **D)** IgA1 MFI, plotted on a log10 axis (1.64-fold, p = 0.014; 8/8 lines); four of the eight lines do not secrete IgA and are included. **E)** CD19 MFI (1.16-fold, p = 0.46; 6/8 lines). Values are the geometric mean ratio (HPA^+^/HPA^-^) with paired, 2-tailed Student’s t-test on log10-transformed values, n = 8.

**Supplementary Table 1.** Demographic and baseline clinical characteristics of IgA nephropathy patients and healthy-control donors.

| **Donor ID** | **Sex** | **Age (yr)** | **Ethnicity/**  **Race** | **SCr (mg/dL)** | **eGFR (mL/min/1.73 m²)** | **UPCR (g/g)** |
| --- | --- | --- | --- | --- | --- | --- |
| IgAN1 | Male | 34 | Non-Hispanic White | 1.9 | 47 | 0.337 |
| IgAN2 | Male | 23 | Non-Hispanic White | 0.8 | 128 | 0.492 |
| IgAN3 | Male | 33 | Non-Hispanic White | 3.5 | 23 | 3.596 |
| IgAN4 | Male | 35 | Non-Hispanic White | 3.7 | 21 | 1.492 |
| IgAN5 | Male | 63 | Non-Hispanic White | 4.1 | 16 | 2.888 |
| IgAN6 | Male | 43 | Non-Hispanic White | 4.9 | 14 | 1.451 |
| IgAN7 | Male | 46 | Non-Hispanic White | 1.5 | 58 | NA (UP<6) |
| IgAN8 | Male | 33 | Non-Hispanic White | 1 | 102 | 0.048 |
| IgAN9 | Male | 43 | Non-Hispanic White | 1.1 | 85 | 0.044 |
| IgAN10 | Female | 47 | Non-Hispanic White | 0.9 | 79 | 0.606 |
| IgAN11 | Female | 35 | Non-Hispanic White | 14.3 | 3 | 0.805 |
| IgAN12 | Male | 73 | Non-Hispanic White | 1.3 | 58 | 0.309 |
| IgAN13 | Female | 56 | Non-Hispanic White | 8.3 | 5 | 1.924 |
| IgAN14 | Male | 54 | Non-Hispanic White | 1.2 | 72 | 0.641 |
| IgAN15 | Female | 41 | Non-Hispanic White | 0.6 | 116 | 1.043 |
| IgAN16 | Male | 44 | Non-Hispanic White | 1.4 | 64 | 0.051 |
| IgAN17 | Male | 37 | Non-Hispanic White | 1.1 | 89 | 1.76 |
| IgAN18 | Male | 38 | Non-Hispanic White | 1.5 | 61 | 2.5 |
| IgAN19 | Male | 41 | Non-Hispanic White | 1.3 | 71 | 0.108 |
| HC1 | Male | 72 | Non-Hispanic White | 0.5 | 108 | 0.388 |
| HC2 | Male | 46 | Non-Hispanic White | 1 | 94 | 0.593 |
| HC3 | Female | 63 | Non-Hispanic White | 1.3 | 46 | ND |
| HC4 | Female | 42 | Non-Hispanic White | 1 | 72 | 0.038 |
| HC5 | Male | 61 | Non-Hispanic White | 1 | 86 | NA (UP<6) |
| HC6 | Male | 36 | Non-Hispanic White | ND | ND | 0.49 |
| HC7 | Female | 37 | Non-Hispanic White | 1.1 | 66 | NA (UP<6) |
| HC8 | Male | 17 | Non-Hispanic White | 0.9 | 128 | 0.012 |
| HC9 | Male | 15 | Non-Hispanic White | ND | ND | 0.014 |
| HC10 | Male | 41 | Non-Hispanic White | 1.1 | 86 | 0.376 |
| HC11 | Male | 47 | Non-Hispanic White | 0.9 | 106 | 0.666 |
| HC12 | Male | 46 | Non-Hispanic White | 1.2 | 76 | NA (UP<6) |

***Abbreviations:*** IgAN, IgA nephropathy; HC, healthy control; SCr, serum creatinine; eGFR, estimated glomerular filtration rate (2021 CKD-EPI equation, without race coefficient); UPCR, urine protein-to-creatinine ratio; UP, urine protein (mg/dL); NA, not applicable; ND, not done.

| **Reagent/Antibody** | **Target** | **Conjugate** | **Catalog #** | **Manufacturer** | **Dilution** |
| --- | --- | --- | --- | --- | --- |
| HPA lectin | Unmodified GalNAc (cell surface) | Alexa Fluor 647 | L32454 | Invitrogen | 1:100 |
| Phospho-NF-κB p65 (S536) monoclonal antibody | p65 NF-κB (phospho-S536) | Unconj. | MA5-15160 (T.849.2) | Invitrogen | 1:1000 |
| NF-κB p65 whole protein recombinant rabbit monoclonal antibody | p65 NF-κB (total protein) | Unconj. | 701079 (4-2H22L23) | Thermo Fisher Scientific | 1:1000 |
| Goat anti-rabbit IgG (H+L) | Rabbit IgG | AF488 | 4050-30-8 | Southern Biotech | 1:1000 |
| SP1 rabbit monoclonal antibody | Human SP1 | Unconj. | 9389S | Cell Signaling | 1:1000 |
| Donkey anti-rabbit IgG | Rabbit IgG (for anti-SP1 antibody) | AlexaFluor-647 | 406414 | BioLegend | 1:1000 |
| DAPI (4’,6-Diamidino-2-Phenylindole, Dilactate) | Nuclear stain | n/a | 50-403-592 | Thermo Fisher Scientific | 1:1000 |
| Mouse monoclonal antibody specific for human IgA1 (clone B3506B4) | Cell surface and total IgA1 | PE | OB9130-09 | Southern Biotech | 1:500 |
| Anti-human CD138 mouse monoclonal antibody | CD138 (Syndecan-1) | PE-Cy7 | 25-1389-42 | Invitrogen | 1:100 |

**Supplementary Table 2:** Flow cytometry reagents used in the methods.
